# Stability of hematological analytes during 48 hours storage at three temperatures using Cell-Dyn hematology analyzer

**DOI:** 10.5937/jomb0-27945

**Published:** 2021-06-05

**Authors:** Ozmen Sevda Unalli, Yesim Ozarda

**Affiliations:** 1 Bursa City Hospital, Department of Medical Biochemistry, Bursa, Turkey; 2 Uludag University, Faculty of Medicine, Department of Medical Biochemistry, Bursa, Turkey

**Keywords:** Complete blood count, temperature, incubation, differential leukocyte count, kompletna krvna slika, temperatura, inkubacija, diferencijalni broj leukocita

## Abstract

**Background:**

The complete blood count (CBC) with differential leukocyte count (DLC) is one of the most common tests requested by physicians. The results of this test are affected by storage temperature and time of incubation. This study was designed to evaluate the stability of hematologic parameters in blood specimens stored for 48 h at three temperatures.

**Methods:**

K2-EDTA - blood was collected from 22 healthy adults. The CBC was performed using a hematology analyser immediately; 0 time point and at 4, 8, 12, 16, 20, 24, and 48 h after storage at 4 °C, 10 °C or 23 °C. Changes in values of CBC parameters from the 0 time point were determined and reported as % of the initial value.

**Results:**

Red blood cells, platelet, hemoglobin, and mean corpuscular hemoglobin were found stable during 48 h storage at 4 °C, 10 °C or 23 °C. Hematocrite and mean corpuscular volume increased, while white blood cells decreased at 48 h when stored at 23 °C. Lymphocytes, neutrophils, eosinophils, and basophils showed significant differences after 12 h of storage at 23 °C.

**Conclusions:**

Red blood cells, platelet, hemoglobin, and mean corpuscular hemoglobin are the only suitable parameters without refrigeration during 24 h storage. When CBC and DLC are performed, 4 °C can be recommended as the most suitable storage temperature for 12 h storage.

## Abstract (sr)

**Uvod:**

Kompletna krvna slika (CBC) sa diferencijalnim brojem leukocita (DLC) jedan je od najčešćih testova koji zahtevaju lekari. Na rezultate ovog testa utiču temperatura skladištenja i vreme inkubacije. Ova studija je osmišljena da proceni stabilnost hematoloških parametara u uzorcima krvi čuvanim 48 sati na tri temperature.

**Metode:**

K2-EDTA - krv je uzeta od 22 zdrave odrasle osobe. Odmah je određena kompletna krvna slika pomoću hematološkog analizatora; nulta vremenska tačka i na 4, 8, 12, 16, 20, 24 i 48 sati nakon skladištenja na 4 °C, 10 °C ili 23 °C. Promene u vrednostima CBC parametara u odnosu na nultu vremensku tačku su utvrđene i navedene kao % od početne vrednosti.

**Rezultati:**

Eritrociti, trombociti, hemoglobin i srednji korpuskularni hemoglobin bili su stabilni tokom 48 sati skladištenja na 4 °C, 10 °C ili 23 °C. Hematokrit i srednja korpuskularna zapremina su se povećavali, dok su se leukociti smanjili tokom 48 sati kada su bili uskladištene na 23 °C. Limfociti, neutrofili, eozinofili i bazofili pokazali su značajne razlike nakon 12 sati skladištenja na 23 °C.

**Zaključak:**

Eritrociti, trombociti, hemoglobin i srednji korpuskularni hemoglobin su jedini pogodni parametri koji se mogu uzeti u obzir bez hlađenja tokom 24-časovnog skladištenja. Kada se rade CBC i DLC, tempe ratura od 4 °C se može preporučiti kao najpogodnija za 12 sati skladištenja.

## Introduction

Pre-analytical errors account for up to 70% of all mistakes made in laboratory diagnostics, most of which arise from problems in patient preparation, sample collection, transportation, and preparation for analysis and storage [1], but not related to the highly standardized analytical process [2]. The complete blood count (CBC), including differential leukocyte count (DLC), is one of the most common and routine hematological laboratory tests requested by physicians. The results of the CBC can be affected by different pre-analytical factors such as temperature and incubation period [3]. Although clinical hematology laboratories equipped with modern automated analyzers are capable of processing large volumes of hematology tests within a short period, delayed sample analysis is not uncommon in the clinical laboratory workflow when samples are transported from other laboratories or health centres to the laboratory, when analysis cannot be readily performed for technical reasons, or when sample needed for re-testing [4]. As a result, testing is often delayed 12 to 24 hours or more after venepuncture. Excessive delays in processing, however, might compromise the reliability of the results rendering worthless all the efforts to guarantee accurate and precise analysis. Several studies have been published about the stability of whole blood specimens for CBC testing, but results from these studies are varying significantly and largely analyserdependent [5] [6] [7] [8] [9] [10]. What we see as the missing point in these studies is that the analyses of different degrees for CBC and DLC storage were not conducted in a single standard study.

Considering the importance of sample stability for generating reliable results for CBC and DLC and lack of consistent data for optimal sample storage temperature and time, this study was designed to test sample stability for CBC and DLC parameters. To determine the effects of storage temperature on the changes of CBC and DLC parameters accurately, we preferred to store our samples at three different temperatures, at 4°C, 10°C, and 23°C for 48 h. Most of the previous studies' blood samples are stored at only one (4°C or room temperature (RT)) or two (4°C and RT) temperatures. In most of the laboratory, the temperature in the cold sample storage room varies between 4-10°C in daily laboratory practice. Furthermore, it is not easy to maintain temperature stabile at 4°C during the transport of blood samples to the centralized laboratories. Thus, it was necessary to test sample stability at 10°C in addition to 4°C. In most of the previous studies, the sample stability was determined usually at few periods (i.e., at 8 h, 24 h, 48 h). To determine the exact time-course of the changes of CBC and DLC parameters, we measured CBC and DLC parameters at 4 h intervals during the first 24 h after a period.

## Materials and Methods

### Subjects and Samples

The study protocol, the contents of the informed consent form, and the general health and lifestyle questionnaire were approved by the Ethics Committee of Uludag University School of Medicine. After providing written informed consent, 22 healthy adults (11 males and 11 females) from the patients aged between 18-50 years were included in the study. A volunteer was considered healthy if a person declared himself or herself to be healthy, and no acute illness took place during the month preceding the venepuncture and further reported absence of diabetes mellitus, renal failure, increased blood pressure, and medication.

### Laboratory Methods

Venous blood samples were drawn from the cubital region using potassium 2 ethylene-diaminetetraacetic acid (K_2_-EDTA) preserved tubes (BD Vacutainer, Becton Dickinson, USA). The samples were analysed in duplicate 15-30 min after vene puncture. To study the effect of storage temperature, one tube was stored at RT, whereas another two tubes were kept refrigerated at 4°C and 10°C. The effect of storage time on CBC parameters was determined by comparing the results at 4, 8, 12, 16, 20, 24, and 48 hours to the 0 time point; 0 TP sample. Measure ment was performed with the Cell-Dyne 3700 hematology analyser (Abbott Diagnostics, IL, USA) delivering a standard package of variables including white blood cell count (WBC), absolute neutrophil count (NEU), absolute lymphocyte count (LYM), absolute monocyte count (MONO), absolute basophil count (BASO), absolute eosinophil count (EOS), red blood cell count (RBC), hemoglobin (Hb), hematocrit (HCT), mean corpuscular volume (MCV), mean corpuscular hemoglobin (MCH), mean corpuscular hemoglobin concentration (MCHC), red cell distribution width (RDW), platelet count (PLT) and mean platelet volume (MPV).

### Quality Control

Internal and external quality controls were also performed in the participating laboratories to monitor the stability of the assay. The analytical coefficient of variation (CVA) was computed for each analyte from the results of repeated measurements of the internal quality control material measured in each laboratory. We compared the results to the analytical performance specification found in the Westgard updated database, which was shared on the website (biologicalvariation.eu) [11]. The desirable limits for between-day and within-day CVAs were set as half of the within-individual CV, as defined by Aarsand and Buoro [12] [13] [14].

### Statistical Analysis

Changes in values of CBC parameters from the 0 TP were determined and reported as % of the initial value. To determine whether the values of hematological parameters observed at 4 h, 8 h, 12 h, 16 h, 20 h, 24 h, and 48 h during storage of blood samples at 4°C, 10°C, or 23°C were different from their initial values (at 0 h, TP), the repeated measures one-way ANOVA was used. When the repeated measures one-way ANOVA showed significant changes, the Tukey test was used to determine p values. Statistical comparisons between the values observed at the same time point in blood samples stored at three different temperatures were made using one-way ANOVA. A value of p < 0.05 was considered statistically significant. Data were given as the mean±standard error of the mean (SEM).

## Results

**Figure 1:**
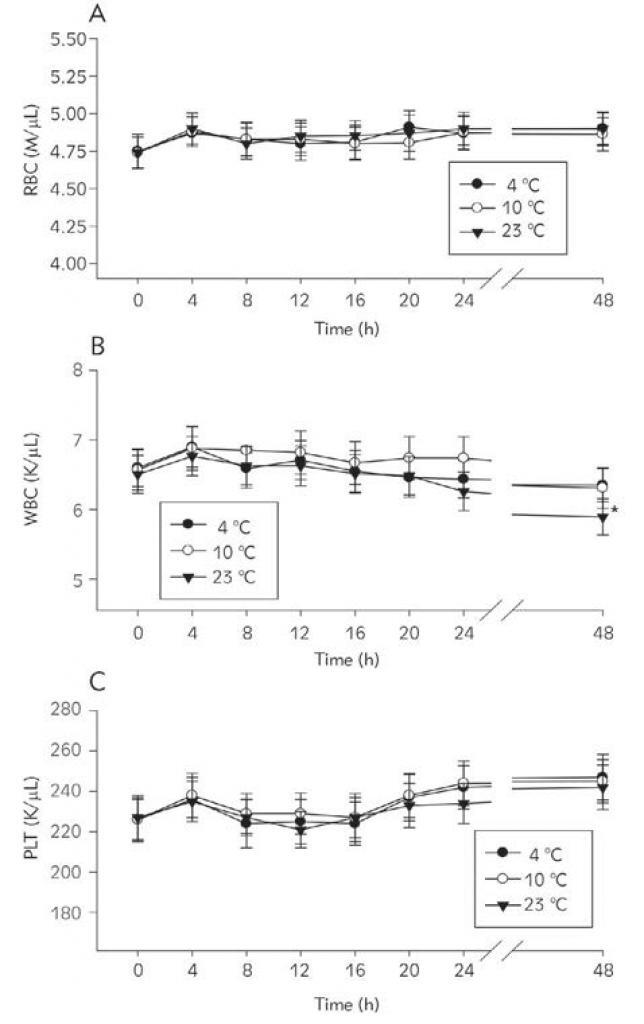
Changes in RBC (A), WBC (B), and PLT (C) with time in blood samples stored at 4 °C, 10 °C, or 23 °C *Different from the initial values (repeated measures one-way ANOVA).

Figure 1 illustrates the changes in mean values of RBC and PLT counts during a 48 h storage period at 4°C, 10°C, or 23°C. It summarizes the percentage of the initial values of RBC, WBC and PLT counts observed at different time points during the 48 h storage period at 4°C, 10°C or 23°C. As seen in Figure 1, RBC and PLT count remained stable. WBC counts remained stable for 48 h at 4°C and 10°C but decreased significantly at 48 h when stored at 23°C (Figure 1B). Statistical analysis with one-way ANOVA revealed significant differences [F(2.63)= 5.237; p < 0.01] in WBC values at 48 h in blood samples stored at the three temperatures.

**Figure 2:**
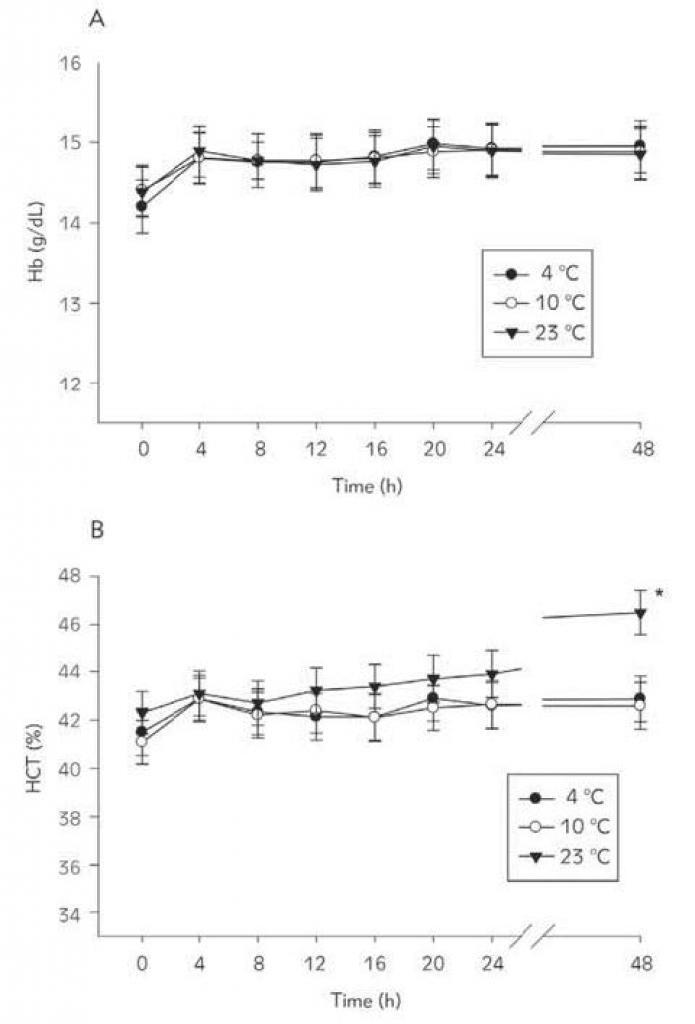
Changes in Hb (A) and HCT (B) with time in blood samples stored at 4 °C, 10 °C, or 23 °C *Different from the initial values (repeated measures one-way ANOVA).

The changes in Hb and HCT values during the 48 h storage period at the three temperatures are shown in Figure 2. It summarizes the percentage of the initial values of Hb and HCT at different time points. The Hb concentrations remained stable during the 48 h storage period at 4°C, 10°C or 23°C (Figure 2A). HCT did not change during the 48 h storage period at 4°C or 10°C (Figure 2B) but increased significantly (Figure 2B; p < 0.001) by approximately 8% from the initial value at 48 h when stored at 23°C. The value of HCT at 48 h in blood samples stored at 23°C was significantly higher [F(2.63) = 5.490, p < 0.01)] than the values observed in blood samples stored at 4 °C or 10 °C. Statistical analysis revealed no significant differences between Hb values observed at 48 h in samples stored at the three temperatures.

Table 1 summarizes the percentage of the initial values of DLC (NEU, LYM, MONO, BASO, and EOS counts) observed at different time points. NEU, MONO, and EOS values remained stable at their initial values during the 48 h storage period at 4°C or 10°C. NEU values decreased significantly at 12-48 h when stored at 23°C. The NEU values at 48 h in blood samples stored at 23°C were significantly lower [F(2.63)= 47.555; p < 0.001] than the values observed for the blood samples stored at 4°C or 10°C. LYM values were stable for 48 h at 4°C, but decreased by approximately 12-15% at 48 h during storage at 10°C, while LYM values increased progressively, by approximately 12-87%, at 12-48 h during storage at 23°C. LYM and NEU values observed for blood samples at 23°C were significantly different from the values observed for blood samples stored at 4°C or 10°C at 16-48 h. BASO values increased gradually and significantly but showed great variations after 12 h during storage at 23°C. EOS values did not change during 48 h storage at 4°C or 10°C but decreased progressively after 8 h during storage at 23°C. MONO values were found to be lower during 48 h storage at 23°C than at 4°C or 10°C. EOS values at 23°C were lower than the values observed for blood samples stored at 4°C or 10°C at 12-48 h. MONO values remained stable during 48 h storage at 4°C or 10°C but decreased slightly at 23°C.

**Table 1:** Changes in NEU, LYM, MONO, EOS, and BASO counts during the 48-hour storage period at 4 °C, 10 °C, or 23 °C NEU, LYM, MONO, BASO, and EOS counts at 0 h were 3.45±0.17 M/ L, 2.33±0.14 K/ L, 0.45±0.03 M/ L, 0.16±0.02 M/ L, and 0.17±0.02 K/ L, respectively. NEU, LYM, MONO, BASO, and EOS counts at 4–48 h were given as a percentage of their respective values at 0 h. Values were given as mean ± SEM (Standard Error of the Mean). *Different from the value at 0 h. #: Different from the values observed at 4 °C or 10 °C for 48 h.

Parameters/Storage time (h)	Storage temperature		
	4 °C	10 °C	23 °C
NEU			
0 h	100.0 ± 0.0	100.0 ± 0.0	100.0 ± 0.0
4 h	105.5 ± 0.9	105.7 ± 0.9	104.3 ± 1.0
8 h	102.9 ± 1.9	106.0 ± 1.0	100.0 ± 1.1
12 h	100.2 ± 1.5	105.1 ± 1.1	92.7 ± 1.9*
16 h	95.9 ± 2.2	104.4 ± 1.1	74.7 ± 3.6*,#
20 h	93.3 ± 2.5	102.2 ± 1.2	53.2 ± 4.8*,#
24 h	99.7 ± 2.8	105.5 ± 1.0	46.8 ± 4.4*,#
48 h	99.6 ± 3.5	103.1 ± 1.4	24.4 ± 2.6*,#
LYM			
0 h	100.0 ± 0.0	100.0 ± 0.0	100.0 ± 0.0
4 h	103.5 ± 1.5	104.2 ± 1.1	105.4 ± 1.2
8 h	103.6 ± 2.2	104.5 ± 1.0	104.1 ± 2.0
12 h	102.2 ± 2.7	100.9 ± 1.4	111.8 ± 2.3*
16 h	102.4 ± 2.7	98.1 ± 1.6	131.5 ± 4.0*,#
20 h	102.4 ± 2.6	98.2 ± 1.7	165.6 ± 7.6*,#
24 h	98.9 ± 2.6	96.7 ± 0.7	171.8 ± 7.8*,#
48 h	95.1 ± 4.4	85.1 ± 2.2*	186.9 ± 12.2*,#
MONO			
0 h	100.0 ± 0.0	100.0 ± 0.0	100.0 ± 0.0
4 h	105.3 ± 3.0	103.7 ± 1.1	106.7 ± 1.5
8 h	101.0 ± 1.1	105.2 ± 1.9	109.1 ± 1.7
12 h	105.2 ± 1.3	104.7 ± 1.7	108.9 ± 3.3
16 h	102.8 ± 1.1	103.0 ± 1.2	110.9 ± 4.3
20 h	103.0 ± 1.3	104.0 ± 1.3	105.3 ± 2.5
24 h	100.8 ± 1.4	104.9 ± 1.2	100.1 ± 1.6
48 h	100.7 ± 1.5	105.9 ± 1.8	88.3 ± 2.5*,#
BASO			
0 h	100.0 ± 0.0	100.0 ± 0.0	100.0 ± 0.0
4 h	94.4 ± 6.6	100.6 ± 6.4	93.1 ± 7.6
8 h	113.5 ±8.9	107.5 ± 9.4	118.0 ± 9.4
12 h	117.0 ± 6.1	106.4 ± 7.4	141.4 ± 22.6*
16 h	119.8 ± 10.0	119.3 ± 9.7	172.5 ± 23.2*,#
20 h	112.6 ± 8.7	117.4 ± 8.4	191.1 ± 25.6*,#
24 h	102.0 ± 6.5	98.9 ± 7.5	261.8 ± 36.0*,#
48 h	88.2 ± 9.2	107.1 ± 9.4	232.6 ± 43.3*, #
EOS			
0 h	100.0 ± 0.0	100.0 ± 0.0	100.0 ± 0.0
4 h	99.6 ± 4.1	104.4 ± 2.6	103.9 ± 5.2
8 h	103.6 ± 3.8	104.0 ± 2.8	91.7 ± 5.2*
12 h	107.5 ± 2.6	105.0 ± 3.4	84.1 ± 4.1*,#
16 h	103.5 ± 3.1	103.4 ± 3.8	68.6 ± 4.8*,#
20 h	102.8 ± 2.8	106.4 ± 3.1	56.6 ± 6.9*,#
24 h	106.3 ± 3.3	110.7 ± 3.5	46.7 ± 5.7*,#
48 h	100.1 ± 4.2	99.9 ± 4.3	33.6 ± 4.7*,#

Table 2 summarizes the percentage of the initial values of MCV, MCH, MCHC, RDW, and MPV at different time points. MCV, MCH, MCHC, and RDW values did not change during the 48 h storage period at 4°C or 10°C. MCV increased significantly at 23°C. MCV values at 48 h in blood samples stored at 23°C were significantly [F(2.63)= 7.01; p < 0.001] higher than the values observed for the blood samples stored at 4°C or 10°C. RDW showed a gradual increase at 12-48 h, while MCHC values decreased gradually at 12-48 h in blood samples stored at 23°C. Statistical analysis revealed that MCHC and RDW values observed in blood samples stored at 23°C for longer than 12 h were significantly different from the values observed in blood samples stored at 4°C or 10°C. MPV remained more or less stable during the 0-16 h storage period but altered significantly during 20-48 h at all three storage temperatures.

**Table 2:** Changes in MCV, MCH, MCHC, RDW, and MPV values during the 48-hour storage period at 4 °C, 10 °C, or 23 °C MCV, MCH, MCHC, RDW and MPV values at 0 °C were 87.7±0.6 fL, 30.5±0.2 pg, 34.7±0.1 g/dL, 15.1±0.2 % and 8.2 ± 0.2 fL, respectively. MCV, MCH, MCHC, RDW, and MPV at 4–48 h were given as a percentage of their respective values at 0 h. Values were given as mean ± SEM (Standard Error of the Mean). *Different from the values at 0 h. #: Different from the values observed at 4 °C or 10 °C for 48 h.

Parameters Storage time (h)	Storage temperature		
	4 °C	10 °C	23 °C
MCV			
0 h	100.0 ± 0.0	100.0 ± 0.0	100.0 ± 0.0
4 h	101.6 ± 0.2	100.2 ± 0.3	100.6 ± 0.2
8 h	99.9 ± 0.2	99.8 ± 0.1	100.9 ± 0.2
12 h	100.0 ± 0.2	100.1 ± 0.2	101.7 ± 0.2
16 h	100.0 ± 0.2	99.9 ± 0.1	102.5 ± 0.2
20 h	99.6 ± 0.2	99.8 ± 0.2	103.9 ± 0.2
24 h	99.6 ± 0.1	99.4 ± 0.4	104.5 ± 0.2
48 h	99.7 ± 0.2	99.8 ± 0.2	108.3 ± 0.3*,#
MCH			
0 h	100.0 ± 0.0	100.0 ± 0.0	100.0 ± 0.0
4 h	99.6 ± 0.3	99.5 ± 0.4	99.3 ± 0.2
8 h	100.0 ± 0.4	100.1 ± 0.4	100.0 ± 0.2
12 h	100.5 ± 0.4	100.0 ± 0.4	99.6 ± 0.2
16 h	101.0 ± 0.5	100.8 ± 0.4	100.1 ± 0.2
20 h	100.1 ± 0.4	101.5 ± 1.4	99.7 ± 0.2
24 h	100.3 ± 0.4	100.8 ± 1.1	100.1 ± 0.5
48 h	101.1 ± 0.4	100.1 ± 0.4	99.2 ± 0.2
MCHC			
0 h	100.0 ± 0.0	100.0 ± 0.0	100.0 ± 0.0
4 h	99.6 ± 0.3	99.3 ± 0.3	98.7 ± 0.3
8 h	100.3 ± 0.3	100.3 ± 0.3	99.3 ± 0.3
12 h	100.8 ± 0.3	100.0 ± 0.3	98.0 ± 0.3*
16 h	101.3 ± 0.4	100.8 ± 0.3	97.7 ± 0.3*
20 h	100.5 ± 0.3	101.8 ± 0.5	96.1 ± 0.3*,#
24 h	100.8 ± 0.3	100.3 ± 0.3	95.3 ± 0.3*,#
48 h	100.3 ± 0.4	101.3 ± 0.4	91.6 ± 0.4*,#
RDW			
0 h	100.0 ± 0.0	100.0 ± 0.0	100.0 ± 0.0
4 h	100.0 ± 0.8	99.4 ± 1.1	101.4 ± 1.0
8 h	99.1 ± 1.3	101.1 ± 1.1	102.6 ± 1.1
12 h	101.8 ± 1.4	99.1 ± 0.9	103.4 ± 1.4*,#
16 h	98.6 ± 1.0	98.9 ± 0.8	106.8 ± 1.2*,#
20 h	99.7 ± 1.0	101.1 ± 1.2	107.0 ± 1.4*,#
24 h	99.9 ± 1.2	99.4 ± 1.1	108.4 ± 1.2*,#
48 h	99.3 ± 1.4	99.6 ± 1.0	114.0 ± 1.1*,#
MPV			
0 h	100.0 ± 0.0	100.0 ± 0.0	100.0 ± 0.0
4 h	100.8 ± 1.3	103.2 ± 1.2	100.7 ± 1.6
8 h	101.8 ± 1.3	104.6 ± 1.4	100.6 ± 1.7
12 h	100.9 ± 1.4	106.6 ± 1.7	103.0 ± 1.7
16 h	103.1 ± 1.1	104.6 ± 1.7	104.2 ± 1.7
20 h	106.1 ± 1.5*	109.9 ± 1.5*	107.0 ± 1.4
24 h	106.7 ± 1.4*	109.5 ± 1.9*	105.5 ± 1.5*
48 h	105.8 ± 1.6*	113.0 ± 2.1*	105.0 ± 2.6*

## Discussion

This study demonstrates that RBC, PLT, Hb, and MCH are stable during 48 h storage at 4°C, 10°C or 23°C. However, HCT and MCV increased, while WBC decreased significantly at 48 h when stored at 23°C. NEU and EOS count decreased, while LYM and BASO count increased after 12 h storage at 23°C. BASO counts showed great variations after 12 h storage at 23°C. WBC, HCT, MCV, MCHC, RDW, NEU, MONO, BASO, and EOS were stable for 48 h if blood samples were stored at 4°C or 10°C. LYM showed a significant difference at 48 h at 10°C and remained more or less stable at 4°C.

Compared to the activities of the testing process, the pre-analytical phase is plagued by a lower degree of standardization, which makes it more vulnerable to errors. Intending to provide guidelines and recommendations, the European Federation of Clinical Chemistry and Laboratory Medicine-Working Group for the Preanalytical Phase (EFLM WG-PRE) issued a survey across laboratories to collect information on pre-analytical approaches. The survey by Cadamuro et al. [15] assessed questions on the willingness of laboratories to engage in pre-analytical analyses. 1265 (94%) responders declared to monitor errors. The interest in pre-analytical issues was high, although substantial heterogeneity was found across European laboratories on pre-analytical phase monitoring. The stability of blood cell counts and related hematological parameters have been investigated in previous studies after storage at 4°C [16] [17], RT [4], and 37°C [18] for 24 h, 48 h, or 72 h using different hematology analyzers [9]. From a review of the literature, it can be said that CBC should be performed as soon as possible after collection, even if a delayed analysis is acceptable for certain parameters and refrigerated storage improves the stability of completed blood cell counts. The data of the current study extend these previous studies by demonstrating the changes in blood cell counts related to hematological parameters at 8 different time points (0, 4, 8, 12, 16, 20, 24, and 48 h) during a 48 h storage period at 4°C, 10°C and 23°C.

In the most recent study, Kayadibi et al. [19] evaluated the stability of CBC in samples containing K 3-EDTA stored at 4°C and RT in upright, horizontal, and upside-down transport positions for up to 240 min by comparing with different stability criteria with 450 samples. Most parameters were stable at all conditions, while just MPV and PDW were unstable at RT in all transport conditions according to the lower and higher CVI, respectively. Buttarello et al. [20] analysed the effect of pre-analytical and analytical variables on MPV. Analyses of the above values were performed in duplicate in 170 healthy adults of both sexes within 30 min from the collection and after 4 hours. A re-analysis was performed for a period of up to 24 hours on samples maintained at RT and 4°C using either K 2-EDTA or Na-citrate as anticoagulants. The stability over time of MPV closely depends on the anticoagulant used, storage temperature, and the time interval between venepuncture and analysis.

In the present study, RBC, WBC, PLT, Hb, MCH, and HCT were observed to remain more or less stable for 48 h around the initial levels with analytically acceptable and clinically negligible changes (< 5%) if blood samples were stored at 4°C or 10°C. More recently, different authors have confirmed that measurements of Hb concentrations and RBC counts are stable up to 72 h after blood collection if blood is refrigerated at around 4°C [17] [21]. This study confirms these findings. On the other hand, WBC counts were seen to decrease and HCT to increase at 48 h if blood samples were stored at 23°C (Figure 1B and Figure 2B). The decrease in WBC counts and the increase in HCT has also been reported in previous reports when blood samples were stored at RT [9] [19]. In agreement with other reports, in the current study, significant increases in MCV and gradual decreases in MCHC were observed at 48 h storage time in blood samples stored at 23°C. Furthermore, there was a significant increase in RDW values at 23°C. The increases in MCV and RDW showed concomitant gradual decreases in MCHC at 12-48 hours at 23°C, but not at 4°C or 10°C. The concomitant changes in MCV, RDW, and MCHC observed in this study are most likely the result of red cell swelling at 23°C as has been suggested in earlier reports [22] [23] [24] [25].

In the present study, MPV was found to be significantly increased during 20-48 h at all three storage temperatures. This finding confirms most previous studies which explain this finding with the result of platelet swelling [10] [17] [19]. Regarding PLT, there are some conflicting results which have pointed out significant changes [17] and no significant changes [10] as were observed in the present study during 48 h storage at 4°C, 10°C or 23°C. However, no significant difference was also noted for PLT in a previous study using the same autoanalyser (Cell Dyn 3500, Abbott) with the impedance method for CBC and the optical method for DLC [4]. Taken together, the results of the current investigation support the conclusions of previous studies that analyte stability on hematological parameters varies not only according to the investigated parameter but also according to storage temperature and the measurement system employed [3] [6] [9].

It has been reported that WBC subpopulations change over time, and incubation at RT resulted in the alteration of certain cell characteristics [6] [10]. Wood et al. [4], using Cell Dyn 3500, also noted a decrease in NEU and an increase in LYM and BASO at 24 h at RT. Gulati et al. [26] used a Coulter GEN-S counter (Beckman Coulter, Hyaleah, FL) and noticed a progressive increase in NEU, LYM, and EOS, and a decrease in MONO during the first 24 h at RT. However, in a previous study using Coulter Maxm (Beckman Coulter, Hyaleah, FL), a decrease in LYM and MONO, and an increase in LYM and EOS were noted [10]. These conflicting results could be attributed to various factors, mainly the measurement analysers used [27]. Besides, the anticoagulant used can cause these different results. Cellular elements are known to have limited stability in blood containing ethylenediamine-tetraacetate (EDTA). K_2_-EDTA-anticoagulated blood is the specimen of choice for automated cell counts, according to the International Council of Haematology [15]. As K_3_-EDTA can ad versely affect some antibodies or assays and K_2_-EDTA is the preferred anticoagulant for hematology measurements [28], the use of the same manufacturer and model of tubes for standardization should be encouraged.

Although laboratory refrigerators are usually set to 4°C (4-8°C), when the follow up for the temperature of the refrigerators is ignored in particular, the inside temperature may vary 4-10°C around the storage sites (in lower cases) due to frequent use of the refrigerators. In practice, laboratory personnel put the samples wherever a place can be found in the refrigerator. Therefore, in this study, the effect of the storage temperature at 10°C in addition to 4°C and 23°C was investigated. The data showed that the changes in hematological parameters were more or less the same when samples were stored at 4°C and 10°C at 24 h. However, a significant alteration was observed only for LYM at 48 h when the samples were stored at 10°C, but not at 4°C. Therefore, it should be suggested to laboratory personnel that they ensure that the CBC samples are stored around 4°C if the samples are waiting, especially more than 24 h.

In conclusion, these data show that the main CBC parameters remain stable during 24 h storage at RT or colder. When CBC and DLC are performed, 4°C can be recommended as the most suitable storage temperature for 12 h storage. In case of storage without refrigeration, samples should be analyzed within 24 h, and the only suitable parameters under these conditions are RBC, PLT, Hb, and MCH. It can be concluded that recommendations should be made that all laboratories that handle a large number of hematology specimens determine the effect of sample storage and storage time to minimize errors.

## Acknowledgments

This study was supported by the Research Fund of Uludag University (UAP(T)-2011/48). We especially thank all the volunteers for their participation in this study.

## Authors’ contributions

All listed authors have made substantial contributions to conception and design, acquisition of data, or analysis and interpretation of data, participating in drafting the manuscript or revising it critically for content, and approval of the final version of the submitted manuscript.

## Conflict of interest statement

The authors reported no conflict of interest regarding the publication of this article.
